# Elucidating the Signal Transduction Mechanism of the Blue-Light-Regulated Photoreceptor YtvA: From Photoactivation to Downstream Regulation

**DOI:** 10.1021/acschembio.3c00722

**Published:** 2024-02-22

**Authors:** YongLe He, Jinnette Tolentino Collado, James N. Iuliano, Helena A. Woroniecka, Christopher R. Hall, Agnieszka A. Gil, Sergey P. Laptenok, Gregory M. Greetham, Boris Illarionov, Adelbert Bacher, Markus Fischer, Jarrod B. French, Andras Lukacs, Stephen R. Meech, Peter J. Tonge

**Affiliations:** † Department of Chemistry, Stony Brook University, Stony Brook, New York 11794, United States; ‡ School of Chemistry, University of East Anglia, Norwich NR4 7TJ, U.K.; § Department of Biophysics, Medical School, University of Pecs, Szigeti ut 12, 7624 Pecs, Hungary; ∥ Central Laser Facility, Research Complex at Harwell, Rutherford Appleton Laboratory, Didcot OX11 0QX, U.K.; ⊥ Institut für Biochemie und Lebensmittelchemie, Universität Hamburg, Grindelallee 117, D-20146 Hamburg, Germany; # TUM School of Natural Sciences, Technical University of Munich, 85747 Garching, Germany; ∇ The Hormel Institute, University of Minnesota, Austin, Minnesota 55912, United States

## Abstract

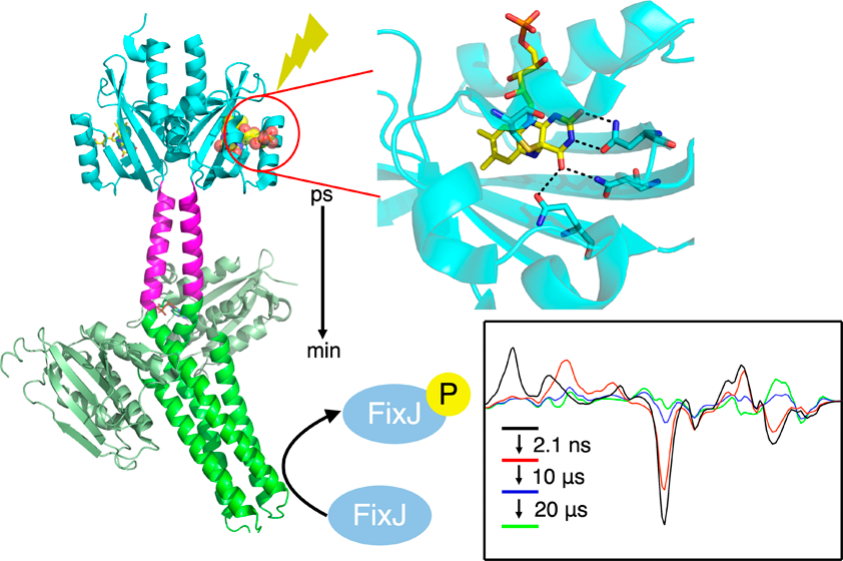

The blue-light photoreceptor YtvA from *Bacillus subtilis* has an N-terminal flavin mononucleotide (FMN)-binding light-oxygen-voltage (LOV) domain that is fused to a C-terminal sulfate transporter and anti-σ factor antagonist (STAS) output domain. To interrogate the signal transduction pathway that leads to photoactivation, the STAS domain was replaced with a histidine kinase, so that photoexcitation of the flavin could be directly correlated with biological activity. N94, a conserved Asn that is hydrogen bonded to the FMN C2=O group, was replaced with Ala, Asp, and Ser residues to explore the role of this residue in triggering the structural dynamics that activate the output domain. Femtosecond to millisecond time-resolved multiple probe spectroscopy coupled with a fluorescence polarization assay revealed that the loss of the hydrogen bond between N94 and the C2=O group decoupled changes in the protein structure from photoexcitation. In addition, alterations in N94 also decreased the stability of the Cys-FMN adduct formed in the light-activated state by up to a factor of ∼25. Collectively, these studies shed light on the role of the hydrogen bonding network in the LOV β-scaffold in signal transduction.

## Introduction

The light-oxygen-voltage (LOV) domain photoreceptors are members of the Per–ARNT–Sim (PAS) protein superfamily, which utilize a noncovalently bound flavin mononucleotide (FMN) cofactor that absorbs light at wavelengths shorter than 500 nm.^1−3^ The LOV photoreceptor contains an output domain that is fused either to the N-terminal A’α helix or C-terminal Jα helix of the LOV domain and controls the activity of the output domain by undergoing a conformational change upon photoexcitation.^4,5^ Whereas LOV domain photochemistry has been extensively studied, the precise details by which ultrafast structural changes in the FMN binding site result in the activation of the output domain remain to be fully elucidated. One reason for this gap in knowledge arises from the relatively few studies that have focused on systems in which output domain activity can be directly correlated with the perturbation in structural dynamics caused by absorption of light by the LOV domain. In the present work, we focus on YF1,^6^ a designed optogenetic construct in which the LOV domain of the photoreceptor YtvA^7^ is fused to a histidine kinase enabling light-induced signal transduction to be analyzed.

**Figure 1:**
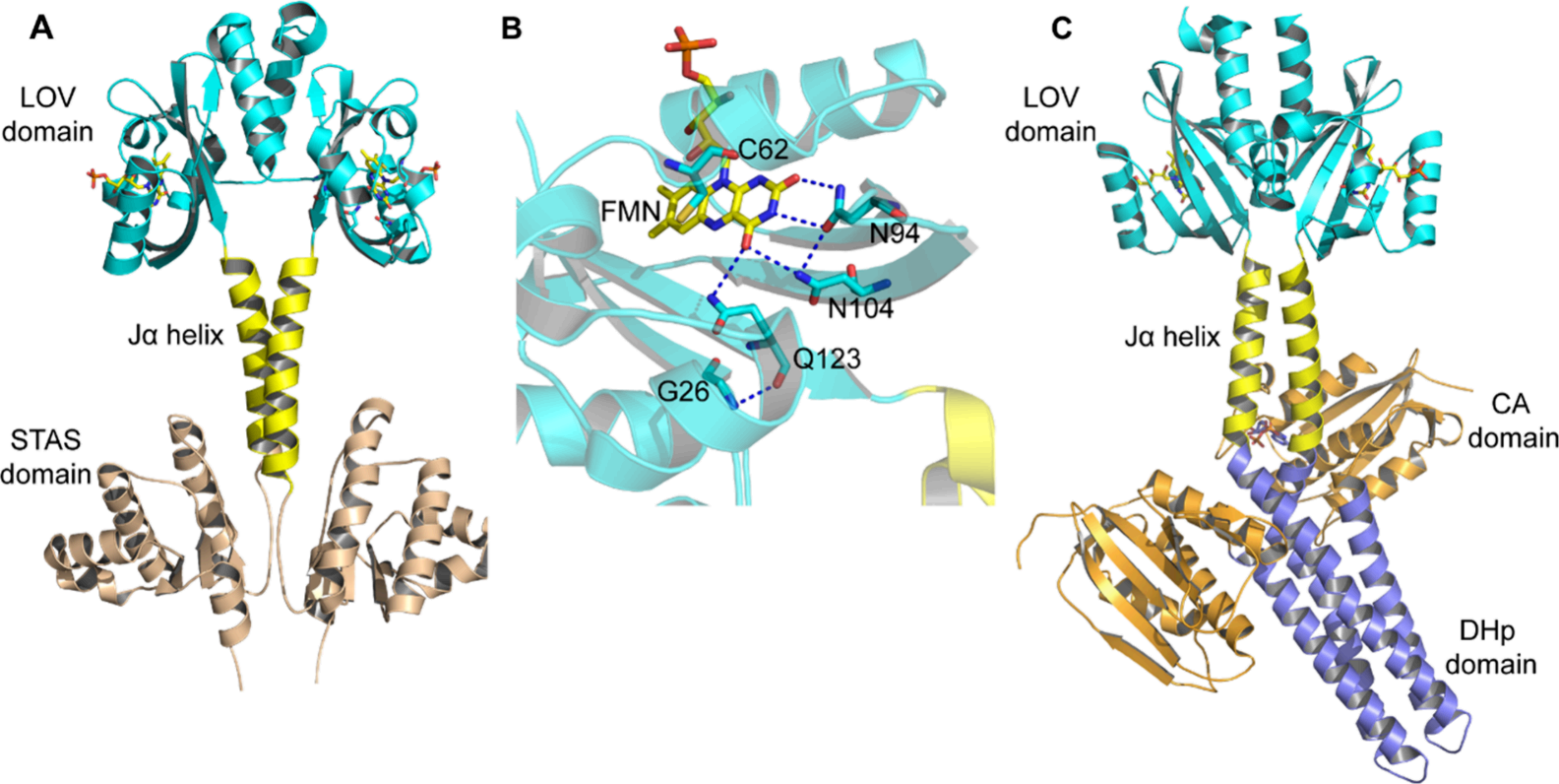
Structures of YtvA and YF1. (A) The solution NMR structure of YtvA (PDB: 2MWG, BMRB: 17643) shows the N-terminal LOV domain (cyan), the C-terminal STAS domain (wheat), and the Jα helix (yellow).^9^ (B) Hydrogen bonding network that connects FMN to the β-sheet scaffold in YtvA. (C) X-ray structure of the optogenetic construct YF1 (PDB: 4GCZ) showing the N-terminal LOV domain (cyan) with the Jα helix (yellow) and the C-terminal FixL histidine kinase composed of the catalytic (CA) domain (orange) and the histidine phosphotransfer domain DHp (slate).^8^ The figures were made using Pymol.^10^

YtvA is a blue-light-regulated transcription factor from *Bacillus subtilis* that is composed of an N-terminal LOV domain fused to a C-terminal sulfate transporter and anti-σ factor antagonist (STAS) domain.^7^ The solution NMR structure of YtvA reveals a head-to-head dimer in which the LOV and STAS domains are connected through two Jα helices that are tilted with respect to each other (Figure 1A).^8,9^ The FMN binding pocket has a similar hydrogen bonding network found in other LOV domain photoreceptors in which the isoalloxazine ring N3, N5, C2=O, and C4=O groups form interactions with conserved Gln and Asn residues.^3^ In YtvA, N94 is hydrogen bonded to C2=O and N3, N104 to C4=O, and Q123 with N5 and C4=O (Figure 1B and Figure S1). In addition, C62, a conserved Cys in the binding pocket, is positioned above the cofactor and forms an adduct with the isoalloxazine C4a in the light state.

Using time-resolved spectroscopy, previous studies showed that the formation of the Cys-FMN-C4a adduct in *As*LOV2, the well-studied LOV domain from *Avena sativa* phototropin 1, is accompanied by the protonation of FMN N5 and rearrangement of the hydrogen bonding network on the microsecond timescale. In *As*LOV2, the network includes N482 (N94 in YtvA), N492 (N104 in YtvA), and Q513 (Q123 in YtvA), and photoactivation involves the rotation of Q513 that alters the conformation of a downstream Asn, N414, leading ultimately to the unfolding of the Jα helix.^11,12^ In YtvA, a similar mechanism is thought to be employed except that photoactivation is proposed to result in rotation of the two Jα helices with respect to each other, thereby modulating the ability of the STAS domain to bind ligands.^13−16^

In this study, we explore the role of conserved Asn (N94) in photoactivation. The LOV domains of YtvA and *As*LOV2 are highly conserved, and N94 in YtvA LOV is expected to link excitation of FMN to the LOV β-sheet by hydrogen bonding the C4=O of the FMN and the downstream Asn residue (N104). N94 has been replaced with Ala, Asp, and Ser (N94A, N94D, and N94S), and time-resolved multiple probe spectroscopy (TRMPS) has been used to elucidate the photoactivation mechanism.^17,18^ In addition, since the functional readout of the STAS domain is difficult to quantify, we have used a construct developed by Möglich and co-workers in which the STAS domain has been replaced with the histidine kinase FixL from *Bradyrhizobium japonicum* (*B. japonicum*) resulting in the light-regulated kinase YF1 (Figure 1C).^6^ YF1 retains the dimer structure found in YtvA and catalyzes the light-dependent phosphorylation of the transcription factor FixJ which *in vivo* was shown to trigger the Fixk2 DNA promoter to initiate gene transcription.^5,8,19^ Here, we used a fluorescence polarization (FP) assay to quantify ADP produced by phosphorylation of FixJ by YF1 and demonstrate that N94 plays a key role in coupling FMN excitation to downstream signaling. In particular, modulating the hydrogen bond between N94 and the isoalloxazine C2=O group decouples the communication between the FMN and the downstream effector domain, which leads to a partial loss of light regulation.

## Results

### Absorption Spectra and Dark-State Recovery of Wild-Type YtvA and N94 Variants

Under constant illumination, the absorbance spectra of both wild-type and mutant YtvA proteins show a characteristic bleach in the 450 nm flavin spectrum and formation of a peak at ∼360 nm, consistent with the formation of the Cys-62/FMN-C4a adduct (Figure S1). As observed previously, the dark state of wild-type YtvA recovers with a time constant of 51 min (Figure S1 and Table 1).^6^ However, the N94 variants show significant differences in the dark-state recovery: N94D YtvA recovers with time constants of ∼10 min, whereas N94A and N94S YtvA recover in ∼2 min. These results indicate that even subtle alterations in the hydrogen bond network destabilize the adduct, in agreement with previous results for the N94D, N94A, and N94S mutants made by Raffelberg et al.^20^ However, while the changes in absorbance at 450 nm report on changes to the isoalloxazine ring of the flavin during the photocycle, the UV–vis spectra contain no information on the accompanying alterations in protein structure that are associated with light absorption.^21,22^ Thus, ultrafast IR spectroscopy was also used to investigate the mechanism of photoactivation.

**Table 1:** Dark-State Recovery of Wild-Type YtvA, YF1, and Mutants

	wild-type	N94A	N94Q	N94D	N94S
YtvA τ (min)^a^	51.2 ± 0.1	1.8 ± 0.1	15.4 ± 0.2	14.5 ± 0.2	2.92 ± 0.02
YF1 τ (min)^a^	50.7 ± 6.7	1.22 ± 0.11	11.0 ± 0.6	12.5 ± 0.5	1.51 ± 0.36

a Time constants were obtained by fitting the change in absorbance at 450 nm to a single exponential equation. The experiment was repeated twice, and the error is the standard deviation of the mean.

### Ultrafast Structural Dynamics of Wild-Type YtvA

**Figure 2:**
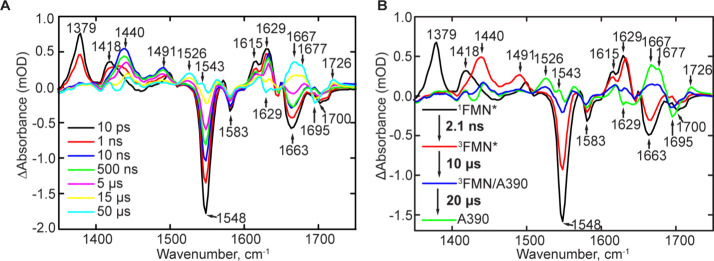
TRMPS spectra of wild-type YtvA. (A) TRMPS spectra selected from 100 fs and 1 ms following excitation at 450 nm. (B) Evolution-associated difference spectra (EADS) obtained by globally fitting the TRMPS data in A to a sequential exponential model ^1^FMN* → ^3^FMN* → ^3^FMN*/A390 → A390.

To provide a foundation for assessing the impact of the N94 mutations on photoreceptor function, we first used time-resolved multiple probe spectroscopy (TRMPS),^17,18^ with a subpicosecond time resolution, to analyze the ultrafast structural dynamics of wild-type YtvA. The temporal evolution of the TRMPS spectra of dark-adapted wild-type YtvA at selected time points is shown in Figure 2. Bleaches (negative signals) are associated with depopulation of the ground or dark-adapted state, while transients (positive signals) are associated with excited-state structural changes induced by the <100 fs excitation pulse (450 nm).

The TRMPS spectra broadly resemble the spectra of other LOV domain photoreceptors and are consistent with our previously published data.^23,24^ Evolution-associated difference spectra were generated by globally fitting the TRMPS spectra to a sequential decay model of 4 components using Glotaran (Figure 2).^25^ This yielded time constants of 2.1 ns and 10 and 20 μs for the decay of ^1^FMN*, ^3^FMN*, and ^3^FMN*/A390, respectively. As described previously by Iuliano et al.,^23^ the first EADS (Figure 2B, black) shows the formation of the singlet excited state (^1^FMN*) formed instantaneously after excitation within the time resolution of the experiment (100 fs) and is characterized by transients at 1379, 1418, 1615, and 1629 cm^–1^ and bleaches at 1548, 1583, 1663, and 1700 cm^–1^. The lower frequency transients (1379 and 1418 cm^–1^) and the bleaches (1548 and 1583 cm^–1^) are assigned to flavin ring vibrational modes in the excited and ground states, respectively, while the higher frequency bleaches and transients from 1600 to 1700 cm^–1^ are assigned to protein modes overlapping with FMN carbonyl vibrations.^26,27^^1^FMN* decays to the triplet excited-state ^3^FMN* through intersystem crossing in 2.1 ns and is associated with the rise of transients at 1440 and 1491 cm^–1^ and a reduction in intensity of the 1548 and 1663 cm^–1^ bleaches (Figure 2B, red).^23,24,28^

The Cys-62/FMN-C4a adduct forms in 10 μs (Figure 2B, blue) and causes dramatic changes in the protein spectrum, which initially contains features from both ^3^FMN* and A390, and then evolves into a spectrum of just A390 over the course of ∼20 μs (Figure 2B, green). Adduct formation is marked by the appearance of a pair of modes at 1543(+)/1548(−) cm^–1^ assigned to C4–C10a vibrations of the isoalloxazine ring, while transients at 1526 and 1667 cm^–1^ are assigned to protein modes.^24^ The high frequency transient at 1726 cm^–1^ is assigned to the flavin C4=O vibration of the light state, which is formed due to the loss of the hydrogen bond from Q123 and/or changes in the electronic structure of the isoalloxazine ring resulting from adduct formation. Bleaches observed at 1629 and 1695 cm^–1^ are assigned to modes arising from the β-sheet and Q123, respectively (Figure 2B, green line).^24,29,30^

### TRMPS Spectral Assignment Using ^15^N-Apoprotein-YtvA and [U-^15^N_4_]FMN YtvA

**Figure 3:**
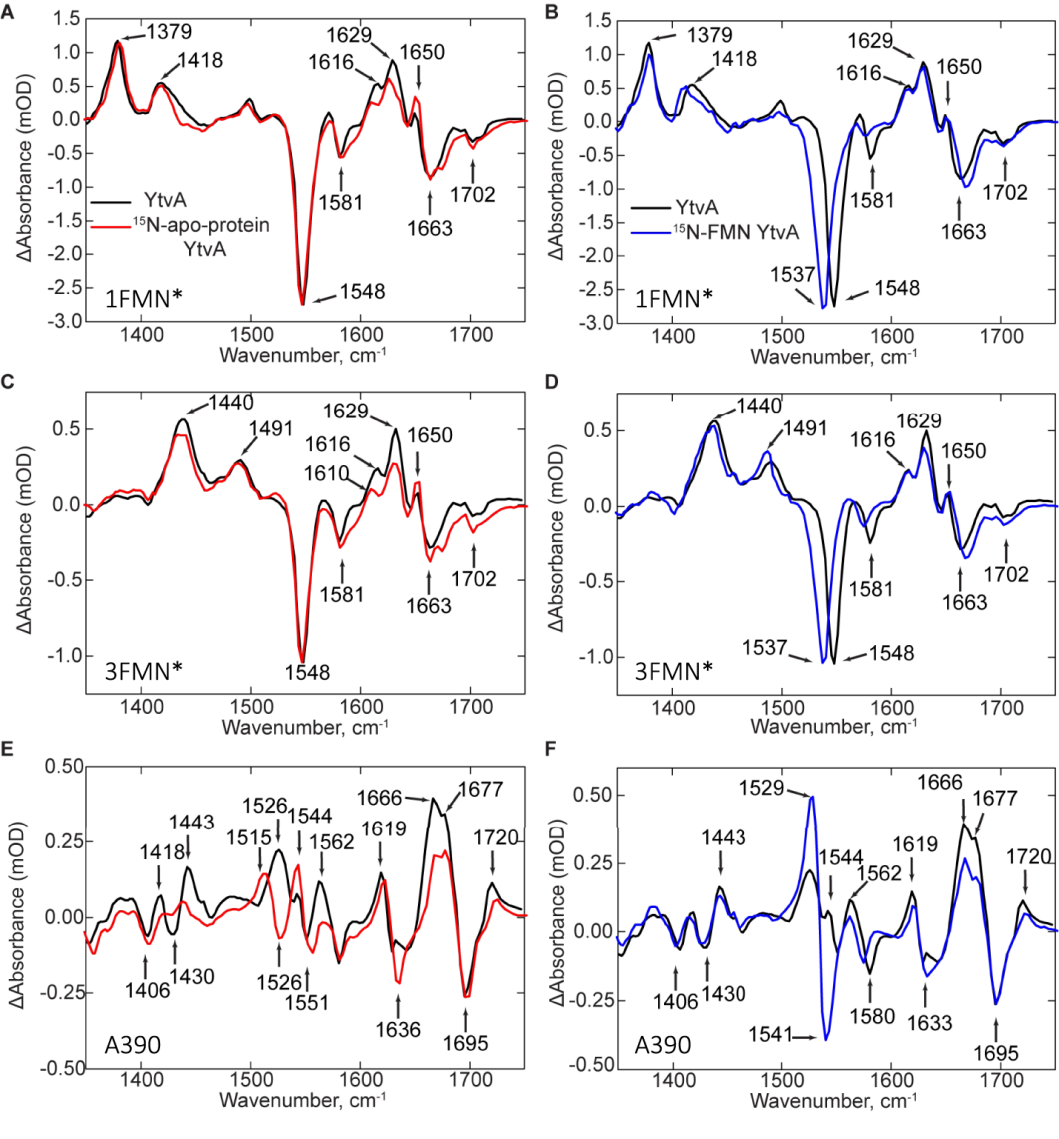
TRMPS spectra of isotope-labeled YtvA. TRMPS spectra of the ^15^N-labeled apo-YtvA (^15^N-apo-YtvA) or [U-^15^N_4_]FMN YtvA. Panels (A), (C), and (E) contain TRMPS spectra of ^1^FMN*, ^3^FMN*, and A390 for ^15^N-apo-YtvA (red), respectively, while panels (B), (D), and (F) are the corresponding spectra for [U-^15^N_4_]FMN YtvA (blue). In each case, the spectra are superimposed on the spectra of nonisotopically labeled YtvA (black).

To provide a more detailed assignment of the EADS for wild-type YtvA, the TRMPS measurement in Figure 2 was repeated with ^15^N-labeled YtvA apoprotein bound to unlabeled FMN (designated as ^15^N-apo-YtvA) and unlabeled apoprotein reconstituted with [U-^15^N_4_]FMN. These samples were prepared using an engineered riboflavin transporter cell line.^31^Figure 3 shows the experimental data for both samples. The spectrum for ^1^FMN* shows the characteristic excited-state transients (1377 and 1418 cm^–1^) and ground-state bleaches (1548 and 1581 cm^–1^), which are unperturbed in the ^15^N-apo-YtvA protein compared to the [U-^15^N_4_]FMN protein sample where the bleaches at 1548 and 1581 cm^–1^ are redshifted to 1537 and 1575 cm^–1^ (Figure 3A,B). The 1548 cm^–1^ mode is assigned to the N5=C4a-C10a=N1 of the isoalloxazine ring of the FMN, while the 1581 cm^–1^ mode is due to the C4a=N5 stretch mode of the isoalloxazine ring. The bleach at 1663 cm^–1^ in wild-type YtvA appears to be a mixture of FMN and protein modes, with a bleach remaining at this position in ^15^N-apo-YtvA, whereas in the [U-^15^N_4_]FMN protein, the 1663 cm^–1^ band is blueshifted to 1672 cm^–1^. Other protein modes in the 1600–1650 cm^–1^ region appear to be redshifted accounting for the decreased intensity of the transients in the spectra of ^15^N-apo-YtvA compared with the [U-^15^N_4_]FMN sample where we do not observe a change in intensity in this region (Figure 3B). The transient at 1650 cm^–1^ did not shift in the ^15^N-labeled apoprotein or [U-^15^N_4_]FMN samples; therefore, it is assigned to the FMN C2=O mode, in agreement with DFT calculations of [U-^15^N_4_]FMN.^24,28,32,33^ The transients at 1629 and 1616 cm^–1^ in the ^15^N-apo-YtvA spectra are both redshifted by 7 cm^–1^ to 1622 and 1609 cm^–1^ but unperturbed by [U-^15^N_4_]FMN labeling (Figure 3B). Thus, these bands could be assigned to vibrational modes of N94 or N104 that form part of the hydrogen bonding network that surrounds the isoalloxazine ring. The 1581 cm^–1^ bleach is redshifted by [U-^15^N_4_]FMN labeling but is not affected by labeling the protein, so this band is assigned to an isoalloxazine ring mode associated with C4a=N5 based on previous studies.^23,24,34^

Figure 3C,D reflects the formation of ^3^FMN* via intersystem crossing and is characterized by a decay of the ^1^FMN* transients (1377 and 1418 cm^–1^) and a rise of transients at 1440 and 1491 cm^–1^. The 1663 cm^–1^ band is split into two bands in the spectrum of ^15^N-labeled protein and blueshifted in the spectrum of [U-^15^N_4_]FMN YtvA, indicating that this band is composed of modes from both the protein and FMN. A plausible assignment for the 1663 cm^–1^ protein mode is to N94 or N104, which are hydrogen bonded FMN.^26^ Other vibrational modes did not change significantly as previously reported for *As*LOV2.^24^^3^FMN* decays in 10 μs, and the A390 signaling state is formed through nonsingle exponential kinetics within 20 μs as previously described (Figure 3E,F).^23^

The ^15^N labeling enables assignment of protein modes in the A390 state previously complicated by large changes in the TRMPS spectra upon adduct formation (Figure 3E,F). A bleach at 1430 cm^–1^ and a transient at 1443 cm^–1^ are assigned to proline modes associated with the changes in the β-sheet.^23,35^ A transient at 1526 cm^–1^ in the spectrum of unlabeled YtvA is shifted to 1515 cm^–1^ upon ^15^N labeling, revealing a bleach at 1526 cm^–1^. The 1526 cm^–1^ transient in the wild type and the 1526 cm^–1^ bleach in the ^15^N-apoprotein are assigned to N–H bend modes of the protein backbone, associated with either the changes in the β-sheet and/or Jα helices, upon formation of the A390 state.^23^ The band shifts caused by isotope labeling enable the assignment of a previously obscured transient–bleach pair at 1544/1551 cm^–1^ to FMN-C4-C10a vibrations directly associated with adduct formation at FMN-C4a. In the amide I region, ^15^N labeling of either the protein or FMN alters the shape of a bleach at ∼1630–1638 cm^–1^, indicating that it is composed of modes from both protein and FMN. A similar vibrational mode at 1626 cm^–1^ was observed during formation of the signaling state in *As*LOV2, which was assigned to disordering of the Jα helix.^12,23^ Here, we hypothesize that the bleach around 1630 cm^–1^ reflects the loss of the coiled-coil structure of the Jα helices due to the structural rearrangement of the β-sheet in the LOV domain core, consistent with the tilting/rotation mechanism proposed for YtvA.^14^ All of the assignments are summarized in Table S1.

### Influence of the N94 Variants on Photoactivation

**Figure 4:**
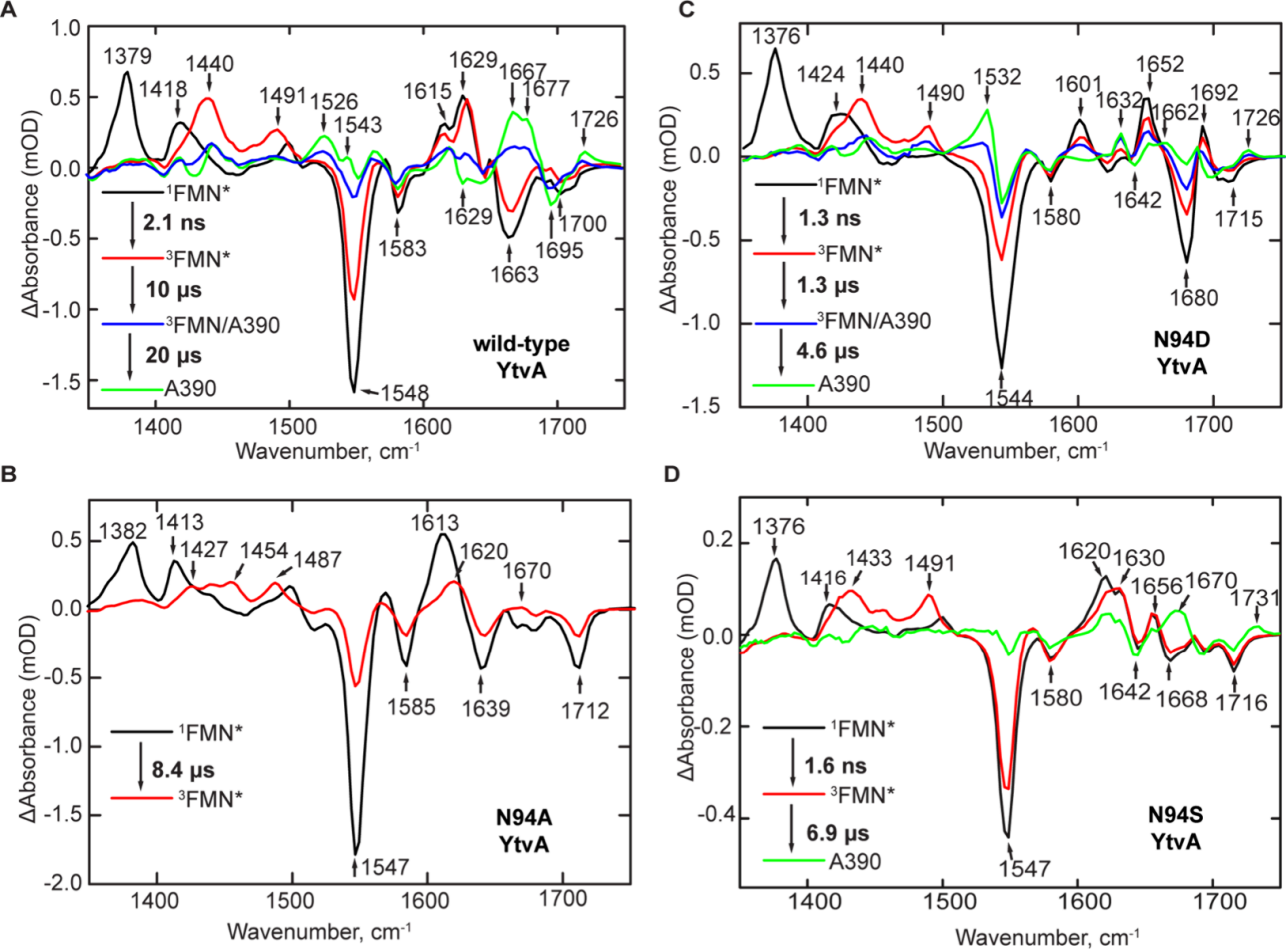
EADS of the N94 YtvA variants. The EADS were obtained by global fitting of the TRMPS data to the sequential kinetic model ^1^FMN* → ^3^FMN → ^3^FMN/A390 → A390. (A) EADS of wild-type YtvA. (B) EADS of N94A YtvA. (C) EADS of N94D YtvA. (D) EADS of N94S YtvA.

The role of the hydrogen bonding network around C2=O in signal transduction was interrogated by making three mutations at residue N94: N94A, N94D, and N94S. The EADS for each of the mutated proteins was extracted from a global fit of the TRMPS data and compared to the EADS of the wild-type YtvA (Figure 4). As shown below, the adduct states of the YtvA N94A and N94S variants cannot be observed in a single-shot laser experiment. Thus, one fewer EADS was needed to fit the data.

#### N94A YtvA

The positions of the flavin bleaches and transients in the TRMPS spectrum of ^1^FMN* N94A YtvA are similar to those observed in wild-type YtvA, except that the 1413 cm^–1^ transient (associated with ^1^FMN*) is redshifted by 5 cm^–1^ in the mutant. In contrast, the N94A mutation has a larger impact on the amide I region of the spectrum. The transient/bleach observed at 1613/1663 cm^–1^ in N94A ^1^FMN*, which is likely due to a combination of protein (amide I) and FMN C2=O vibrational modes, is lacking the doublet at 1615/1629 cm^–1^ and is shifted from the bleach at 1663 cm^–1^ observed in wild-type YtvA (black spectrum in Figure 4A,B). The bleach at 1700 cm^–1^, assigned to Q123 in wild-type YtvA, is shifted by ∼12 cm^–1^ to 1712 cm^–1^ in the N94A variant due to the loss of the hydrogen bond between A94 and N104 (black spectrum in Figure 4A,B). Furthermore, ^1^FMN* decays more slowly to ^3^FMN* than in wild-type YtvA, with a time constant of 3.4 ns compared with 2.1 ns. This suggests that the mutation has induced a change in the interaction of ^1^FMN* and the binding site, slightly increasing the fluorescence lifetime.

The shapes of the transients assigned to ^3^FMN* ring modes (1440 and 1491 cm^–1^ in wild-type) are significantly perturbed by the Asn to Ala replacement, resulting in a broad transient with two peaks at 1427 and 1454 cm^–1^ and a higher frequency transient at 1487 cm^–1^ (Figure 4B, red line). We also observe a 7 cm^–1^ shift in the protein/C2=O transient from 1613 to 1620 cm^–1^, which is not as prominent in the second EADS of wild-type YtvA. The ^3^FMN* shows biphasic decay, with an 8.3 μs component (red EADS) and a long component (blue EADS), that does not decay within the 1 ms timescale of the experiment. The EADS in N94A (Figure 4B, blue spectrum) shows a small rise around 1670 cm^–1^ where marker bands for adduct formation are found in the wild type as well as other LOV domain proteins. However, we do not observe a further evolution of the spectrum to adduct formation in the TRMPS experiment for N94A, consistent with previous reports.^20,23,27^

#### N94D YtvA

N94D YtvA shows the most similarity to wild-type YtvA as adduct formation, and some structural dynamics are preserved (Figure 4C). The first EADS shows the instantaneously formed ^1^FMN* excited state, with transients at 1376 and 1424 cm^–1^ and a bleach at 1544 cm^–1^, which are assigned to C–N vibrations from the isoalloxazine ring in the excited state and ground state, respectively. The protein modes previously assigned from ^15^N-apoprotein labeling are shifted significantly in the N94D variant compared to wild-type YtvA (Figure 4C, black spectrum). Transients observed at 1601 and 1652 cm^–1^ in N94D YtvA are shifted from 1615 (−14 cm^–1^) and 1629 cm^–1^ (+23 cm^–1^). The bleach at 1663 cm^–1^ in wild-type YtvA is shifted by 17 cm^–1^ to 1680 cm^–1^ in N94D YtvA. The 1629 and 1663 cm^–1^ bands are assigned to vibrational modes arising from the side chains of N94 and/or N104, while the transient/bleach pair at 1652/1680 cm^–1^ is assigned to a protein mode. This assignment is based on ^15^N-apo-N94D YtvA showing a blueshift at 1652 cm^–1^ and a redshift at 1680 cm^–1^ upon the ^15^N labeling disrupting the hydrogen bond network between the C2=O, D94, and N104 (Figure S2).^36^ Additionally, a new transient appears at 1692 cm^–1^, and the bleach at 1700 cm^–1^ assigned to Q123 is shifted to 1715 cm^–1^ in N94D YtvA.

^1^FMN* decays to ^3^FMN* in 1.3 ns giving rise to transients at 1440 and 1490 cm^–1^ and a reduction in intensity of the transients at 1376 and 1424 cm^–1^. ^3^FMN* then decays in 1.3 μs, and the Cys adduct is formed through dispersive kinetics giving rise to a spectrum that contains features previously assigned to both ^3^FMN* and A390 and characterized by a reduction in intensity of the transients at 1440 and 1490 cm^–1^ (Figure 4C, blue line). The final adduct spectrum is formed in 4.6 μs and is characterized by a transient/bleach feature at 1532 (+)/1544 (−) cm^–1^ assigned to C4–C10a vibrations from the isoalloxazine ring and some protein contributions based on the ^15^N labeling spectra (Figure 3E,F). In addition, ^15^N labeling of N94D YtvA causes a blueshift in the 1532 cm^–1^ band, suggesting that this band contains both FMN and protein modes (^15^N-apo-N94D YtvA, Figure S2). Adduct formation was accelerated to 4.5 μs compared with the wild type and gives rise to transients at 1632, 1662, and 1726 cm^–1^ assigned to the β-sheet, protein modes, and C4=O, respectively. Bleaches are observed at 1642 and 1695 cm^–1^ and are assigned to the β-sheet and Q123, respectively. When compared to that of wild-type YtvA, the C4–C10a vibration is much more intense in N94D YtvA and the protein mode at ∼1665 cm^–1^ is attenuated. In addition, the transient at 1526 cm^–1^, assigned to β-sheet N–H bending modes in wild-type YtvA, is not present in N94D YtvA. Collectively, the data suggest that although adduct formation is accelerated compared to that in wild-type YtvA, the resulting overall structural changes are more modest in the N94D mutant. This observation is consistent with a previous study in which the N94A mutant promoted faster decay of the triplet state and reduced quantum yield for formation of the adduct.^20^

#### N94S YtvA

In N94S YtvA, the first EADS illustrates the instantaneous appearance of ^1^FMN*, which is characterized by transients at 1379 and 1424 cm^–1^ and bleaches at 1548 and 1583 cm^–1^ (Figure 4D). The transients observed at 1620 and 1630 cm^–1^ are similar to the protein modes at 1615 and 1629 cm^–1^ in wild-type YtvA, while the bleach at 1663 cm^–1^ in wild-type YtvA is shifted by 5 cm^–1^ to 1668 cm^–1^ in N94S YtvA. The similarity in the vibrational spectra of wild-type and N94S YtvA suggests that introduction of S94 does not significantly alter the hydrogen bond network around the C2=O of the FMN. However, the bleach at 1695 cm^–1^ assigned to Q123 is shifted to 1716 cm^–1^ in N94S YtvA, suggesting that the hydrogen bond between Q123 and FMN has been weakened.

^1^FMN* decays to ^3^FMN* in 1.6 ns giving rise to transients at 1433 and 1491 cm^–1^ and depletion of transients at 1376 and 1424 cm^–1^ as in the wild-type YtvA. ^3^FMN* decays in 6.9 μs to a final state that does not closely resemble the A390 state observed in the other proteins. For instance, a weak bleach is observed at 1551 cm^–1^, which can be assigned to the C4–C10a vibration of the isoalloxazine ring, while transients at 1670 and 1731 cm^–1^ are assigned to protein modes and C4=O, respectively. In addition, bleaches observed at 1642 and 1716 cm^–1^ are assigned to the β-sheet and Q123, respectively. In addition, while bleaches at ∼1667 and 1642 cm^–1^ are observed, consistent with changes in the protein structure caused by adduct formation, the 1543 cm^–1^ mode that corresponds to the C4–C10a vibrational mode and the transient at 1526 cm^–1^ assigned to β-sheet N–H bending modes in the wild-type are not observed in N94S YtvA.

### Impact of N94 Mutagenesis on Light-Regulated Kinase Activity

The activity of the YtvA system was studied using the engineered optogenetic tool, YF1, in which the YtvA LOV domain is fused to the histidine kinase from the oxygen sensor FixL.^6^ In both YtvA and YF1, the LOV domain and output domains are connected by a Jα helix that forms a coiled coil in the dimeric protein, while an N-terminal A’α helix also forms a coiled coil. Detailed studies on YF1 support a photoactivation mechanism in which the two LOV domains rotate by 15° leading to a supertwist of the C-terminal Jα helix coiled coil.^8^ A similar reorientation of the two LOV domains in YtvA is supported by structural studies of the isolated LOV domain^13^ and the NMR solution structure.^9^

We first showed that the YF1 system behaves similarly to YtvA. Constant illumination of wild-type and mutant YF1 resulted in a bleach in the 450 nm flavin absorbance (Figure S3), and in each case, the dark state recovered at similar rates to those observed for YtvA (Table 1). We also analyzed the steady-state IR difference spectra of wild-type and mutant YtvA as well as the corresponding YF1 proteins, which revealed that YtvA and YF1 produced similar difference spectra under constant illumination (Figures S4 and S5).

**Figure 5:**
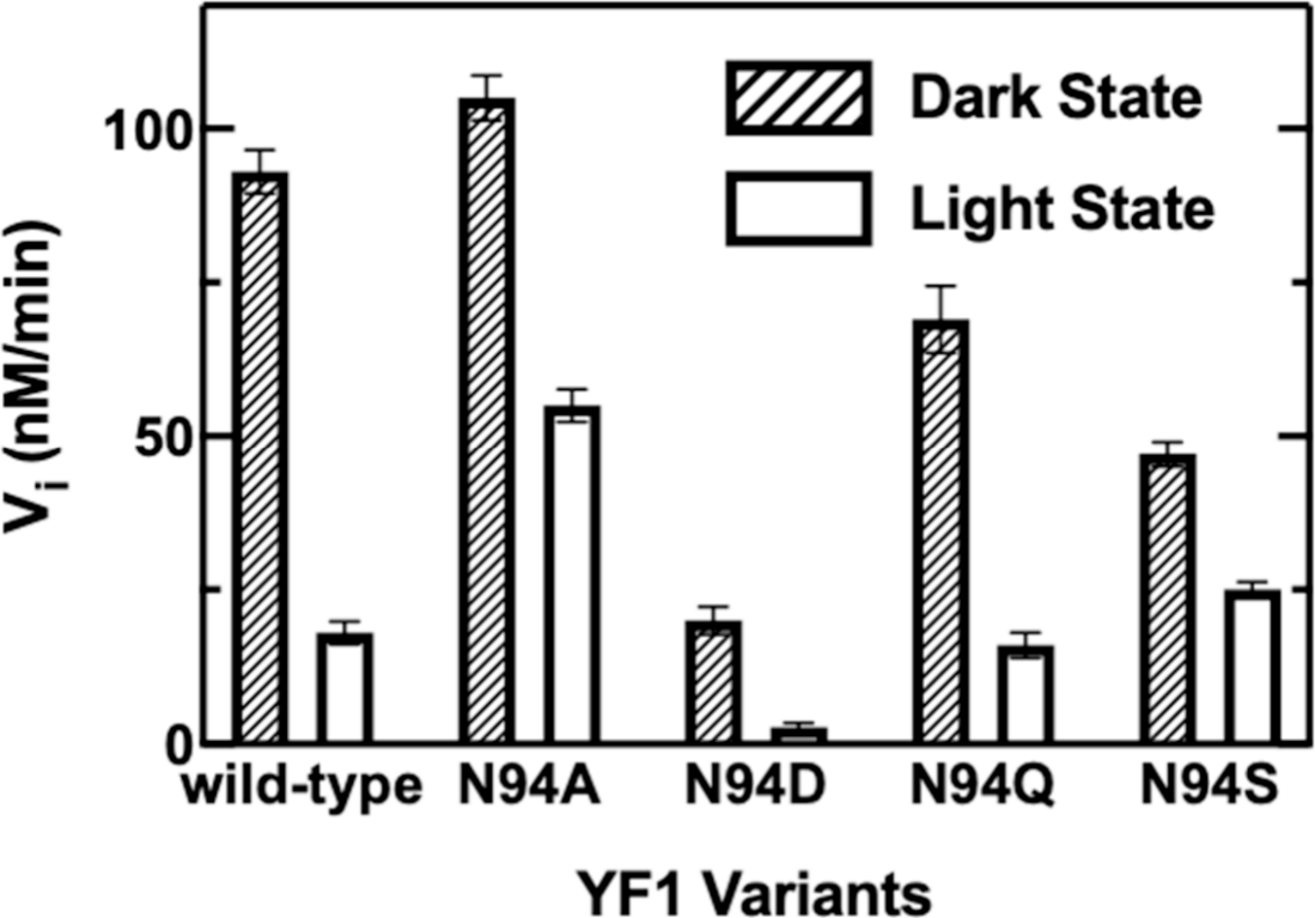
Kinase activity of wild-type and N94 mutant YF1. The kinase activity was measured using the ADP fluorescence polarization assay either in the dark or during constant blue-light irradiation. The change in activity for each protein on illumination is summarized in Table 2. Experiments were repeated at least twice, and the error is the standard deviation of the mean.

In YF1, two reactions take place where first the autophosphorylation of the H161 side chain occurs, and then, a phosphate is transferred from the YF1 H161 to the D55 side chain of the response regulator FixJ.^37^ The autophosphorylation of YF1 is stimulated by the presence of FixJ, even though FixJ does not directly participate in the autophosphorylation reaction (Figure S6A).^38^ The kinase activity of YF1 was consequently measured in the presence of the downstream phosphate acceptor FixJ by quantifying the production of ADP using a fluorescence polarization (FP) assay (Figure S6B).^39,40^ The FP assay uses the Transcreener technology consisting of an antibody selective to ADP over ATP and a far-red fluorescent tracer. ADP produced in the reaction competes with the tracer, changing the fluorescent polarization. The reaction was first optimized by measuring the formation of ADP at different concentrations of YF1 and FixJ in the presence of either 10 or 100 μM ATP. A 1 to 5 ratio of YF1 to FixJ (125 and 625 nM, respectively) was found to generate a robust change in fluorescence polarization upon ADP production in 10 min using 10 μM ATP. The initial velocity for each protein was then determined in the dark and under blue-light illumination (Figure S6B, Figure 5, and Table 2). Upon constant illumination, wild-type YF1 exhibits an ∼5-fold decrease in the rate of ADP production. The N94D and N94Q variants show similarly robust changes in activity upon illumination, although the dark-state kinase activity of N94D YF1 is 5-fold lower than that of the wild type. In contrast, the kinase activity of both N94A and N94S YF1 is reduced by less than a factor of 2 upon blue-light illumination. These results are in general agreement with previous studies by Diensthuber et al. who analyzed the impact of the mutations on YF1 activity using a light-regulated gene expression assay in *Escherichia coli* (*E. coli*).^41^ In the latter work, it was shown that N94A and N94S YF1 were unable to regulate gene expression, while the dark-state gene expression of the N94D variant was suppressed by ∼2-fold compared with that of wild-type YF1. In our studies, we observed a 2-fold change in kinase activity for N94A and N94S and a 5-fold change for N94D, which follows the same trend as the activities determined using the gene expression assay.

**Table 2:** Kinase Activity of Wild-Type YtvA and the N94 Variants^a^

YF1	wild-type	N94A	N94D	N94Q	N94S
ν_i_ dark (nM/min)	93 ± 3	105 ± 3	20 ± 2	69 ± 5	47 ± 2
ν_i_ light (nM/min)	18 ± 1	55 ± 2	2.8 ± 0.6	16 ± 2	25 ± 2
fold change in activity on illumination	5×	1.9×	7×	4×	1.9×

a The initial velocity for ADP production was quantified using the fluorescence polarization either in the dark or on constant blue-light illumination. Experiments were repeated at least twice, and the error is the standard deviation of the mean.

## Discussion

Most of the work performed on LOV domain photoreceptors has focused on exploring the role of adduct formation in the light state.^42^ Our molecular dynamics (MD) simulations on *As*LOV2 showed that Cys-FMN adduct formation leads to disruption of the hydrogen bonds between two conserved Asn residues (N482 and N492) and the flavin C2=O and C4=O groups.^12^ The structural dynamics of the conserved Asn residues is believed to couple the signaling from the excited flavin to the effector domain by modulating the conformation of a downstream Gln residue (Q513).^43^ In the present work, we have extended our previous studies on *As*LOV2 and here focus on the role of N94 in the photoactivation of the LOV domain photoreceptor YtvA (N482 in *As*LOV2). This was accomplished by replacing N94 with Ala, Ser, and Asp residues and analyzing photoactivation over 10 decades of time from picoseconds to μs using time-resolved multiple probe spectroscopy where assignment of the TRMPS difference spectra was aided by uniform ^15^N labeling of either the protein or chromophore. The impact of mutating N94 on biological activity was then assessed using YF1, a construct in which the YtvA LOV domain is fused to a histidine kinase, providing insight into the key role that N94 plays in photoactivation.

### Impact of N94 Mutagenesis on Light-State Formation and Cys-FMN Adduct Stability

Constant illumination of wild-type YtvA and YF1 as well as the N94 variants leads to bleaching of the FMN absorbance at 450 nm, consistent with the formation of the Cys-FMN adduct. Dark-state recovery for wild-type YtvA and YF1 occurs with similar time constants of 51 and 57 min, respectively. For the N94D, dark-state recovery is ∼5-fold faster, whereas it is 20- to 50-fold faster for N94A and N94S. Thus, alteration in the hydrogen bonds to the FMN has destabilized the light state. These observations can be compared with the analysis of photoactivation by TRMPS. Specifically, whereas light-state formation is observed for N94D YtvA, IR bands associated with formation of the Cys-FMN adduct cannot be observed for N94A and N94S YtvA. We speculate that the instability of the Cys-FMN adduct in N94A and N94S results in a very low yield of the light state in the single-shot TRMPS measurements. This conclusion is substantiated by the similarity of the steady-state IR difference spectra for wild-type and mutant proteins under constant illumination (Figures S4 and S5).

The solvent isotope effect (SIE) on the dark-state recovery of other LOV photoreceptors indicates that the rate-limiting step of the adduct decay involves the deprotonation of the flavin N5.^44^ Thus, the difference in thermal recovery rates for the N94 variants in YtvA supports a model in which the hydrogen bond between N94 and C2=O stabilizes the protonated state of N5. In support of this hypothesis, earlier studies on flavoproteins showed that hydrogen bonding to O2, N3, and O4 is a key factor in catalytic activity and increases the ability of N5 to accept a hydride.^45,46^ In addition, the MD simulations on *As*LOV2 indicate that rotation of the Gln side chain out of the FMN binding site is promoted by the rearrangement of the hydrogen bonding network in the β-sheet scaffold. Thus, replacement of N94 with residues that cannot hydrogen bond to the flavin will lead to the Gln side chain remaining in the FMN binding pocket, which promotes cleavage of the Cys-adduct bond.^20^

### The N94 Residue Links FMN Excitation to the LOV β-Sheet

Wild-type YF1 exhibits a 5-fold decrease in kinase activity upon photoactivation. This change in activity is comparable to the ∼10-fold difference observed for YF1 using a cell-based DsRed expression system.^38^ The alteration in output domain activity may appear modest but is similar to those reported in other systems such as the Cry2 Tyr kinase and LOV-regulated Ser/Thr kinase, which exhibit 5- and 10-fold changes in activity upon illumination, respectively.^47,48^ Given the relatively modest dynamic range in wild-type YF1, the observation that N94A YF1 exhibits only an ∼ 2-fold change in activity is significant and indicates that the loss of the hydrogen bond between N94 and C2=O has partially disabled signal transduction from the cofactor binding pocket. The dark-state activity of N94A YF1 is similar to that of the wild type, and thus, the structure of the dark state has not been impacted by the Asn to Ala substitution. The N94S mutant has a similarly small change in light-regulated activity, and we note that both N94A and N94S lead to the biggest change in the stability of the Cys-FMN adduct. Thus, we can conclude that the ability of the protein to stabilize the light state is linked in a fundamental way to the regulation of output domain activity.

Not surprisingly, N94Q YF1 most closely resembles the wild-type photoreceptor and shows a similar (4-fold) change in activity on irradiation, whereas replacement of N94 with Asp reduces the dark-state activity of YF1 to that observed for the light state of the wild-type photoreceptor. However, N94D retains full light regulation, where irradiation results in a further 7-fold decrease in activity. Compared to the wild type, the TRMPS difference spectra of the N94D variant show that structural changes accompanying irradiation are attenuated, consistent with N94D YtvA adopting a signaling state conformation in the dark.

### The Role of the β-Scaffold Hydrogen Bonding Network in Light Regulation

The signal transduction mechanism in the β-sheet scaffold should be consistent with most of the LOV photoreceptors that contain a conserved hydrogen bonding network. However, variation is observed in how photoactivation alters the structural dynamics of the Jα helix.^14^ NMR and CD studies on *As*LOV2 reveal a fluctuation in the β-sheet scaffold upon illumination that results in an unfolding of the Jα helix.^11^ In YtvA, structural studies show that the Jα helix adopts a coiled-coil motif with the solvent-exposed β-scaffold at the dimer interface,^9,13^ where the light-induced structural change is proposed to rotate the Jα helix by ∼5° to regulate the biological activity.^49,50^ The structural dynamics of the YtvA system can be related to the hydrogen bond rearrangement in the β-scaffold and formation of the adduct during photoactivation. Removal of the hydrogen bond between the FMN and residue 94 in the N94A and S variants results in a reduction in light regulation, implying that N94 acts to stabilize the β-sheet structure that is involved in the dimer–dimer interface. In contrast, we propose that D94 would be negatively charged, and this would interfere with the environment around the C2=O of the FMN such that the β-scaffold would adopt a light-adapted structure independent of flavin excitation.

## Conclusions

Multiple approaches were utilized to elucidate the signaling mechanism of YtvA. TRMPS spectroscopy was used to probe the structural changes during the ultrafast photoactivation, and isotope labeling enabled unambiguous assignment of the protein vibrational modes affected by A390 formation. Using site-directed mutagenesis, hydrogen bonds involving the conserved N94 residue were perturbed to explore the role of this residue in photoactivation. The TRMPS spectra of the N94D variant reveal that protein structural changes are attenuated due to the mutation, while in the N94A variant, formation of the A390 state is decoupled from the structural changes observed in the wild-type photoreceptor. The spectral data are complemented by data from an activity assay in which the YtvA LOV domain has been fused to a histidine kinase. The N94D YF1 variant has dark-state activity that resembles the light-state activity of the wild type, while N94A exhibits partial light regulation. In addition, the adduct decay rate can be tuned by changing the hydrogen bonding interactions involving N94, and a correlation is observed between the adduct stability and the light-induced change in kinase activity. Collectively, the results support a mechanism in which the hydrogen bonding network in the β-scaffold transmits ultrafast excitation of FMN to movement of the Jα helix on the μs–ms timescale, thereby driving the biological response caused by photoactivation.

## Methods

### Cloning and Site-Directed Mutagenesis

The pET41-YF1 plasmids containing the YtvA LOV gene from *Bacillus subtilis* (residue 8–126) and the FixL gene from *Bradyrhizobium japonicum* (residue 127–372) were gifts from Dr. Möglich. The gene for full-length YtvA was cloned into a pET15b vector (Novagen) in the frame with an N-terminal six-His tag. The gene encoding *B. japonicum* FixJ was synthesized and cloned into a pET28c vector (GenScript). The N94A, S, Q, and D mutations in pET15b YtvA and pET41c YF1 were generated using QuickChange mutagenesis and the KOD HotStart polymerase (Novagen).

### Expression and Purification of YtvA, YF1, and FixJ

Wild-type and mutant YtvA, YF1, and FixJ were overexpressed in BL21 (DE3) *Escherichia coli* cells. BL21 (DE3) cells were transformed with vectors encoding the respective wild-type or mutant proteins, which were then plated on LB-agar plates containing 50 μg/mL kanamycin for pET41c YF1 and pET28c FixJ or 100 μg/mL ampicillin for pET15b YtvA. A single colony was used to inoculate 10 mL of 2x-YT media (Fisher Bioreagents, BP9743-5) containing 50 μg/mL kanamycin or 100 μg/mL ampicillin, which was shaken overnight at 37 °C (250 rpm). Subsequently, the 10 mL overnight culture was used to inoculate 1 L of 2x-YT media containing the appropriate antibiotic in a 4 L flask, which was then shaken at 37 °C (250 rpm) until the optical density (OD_600_) reached ∼0.4–0.6. The temperature was lowered to 18 °C, and 1 mM isopropyl β-d-1-thiogalactopyranoside (IPTG, Gold Biosciences) was added to induce protein expression. The cells were harvested after 16 h by centrifugation at 5000 rpm (6,238*g*; 4 °C) for 20 min, and the cell pellet was stored at −20 °C until it was needed.

The cell pellet containing each protein was thawed and resuspended in 40 mL of lysis buffer (20 mM Tris (pH 8 × 8), 150 mM NaCl, and 5 mM imidazole) and lysed by sonication. The cell debris was removed by ultracentrifugation at 40,000 rpm (185,511*g*) for 1 h at 4 °C. Each protein sample was purified using a method previously reported.^23^ Briefly, the supernatant was loaded onto a 5 mL Ni-NTA column (GE), which was then washed with 10–20 mL column volumes of lysis buffer containing 10–20 mM imidazole. Chromatography was performed with a gradient of 20 to 500 mM imidazole in lysis buffer leading to elution of the protein at 250 mM imidazole. Fractions containing protein were then pooled and loaded onto a HiLoad 16/600 Superdex-200 column equilibrated with lysis buffer (AKTA purifier). Protein fractions were collected, and the purity of the protein was shown to be >95% by SDS-PAGE. The concentration of YtvA, YF1, and the N94 variants was determined by absorbance spectroscopy using the FMN extinction coefficient (ε_450_ = 12,200 M^–1^ cm^–1^), while the concentration of FixJ was determined using ε_280_ = 4860 M^–1^ cm^–1^. Ethylene glycol (5%) was added to the solutions of the purified proteins in lysis buffer, which were flash frozen with liquid nitrogen and stored at −80 °C or lyophilized and resuspended in D_2_O for the FTIR or TRMPS measurements.

### Isotope Labeling for Apo-YtvA and FMN YtvA

^15^N-labeled apo-YtvA and FMN YtvA were produced using a previously described method.^24^ Samples were produced using a recombinant *E. coli* strain that contained a plasmid directing the low-level expression of a bacterial riboflavin transporter and a second plasmid directing the high-level expression of YtvA. To produce proteins carrying labeled FMN, the strain was cultured with a supplement (7 mg L^–1^) of [U-^15^N_4_] riboflavin. To produce [apoprotein-U-^15^N]-YtvA, the recombinant *E. coli* strain was grown with ^15^NH_4_Cl as the exclusive nitrogen source; unlabeled riboflavin was added to the culture medium at a concentration of 7 mg L^–1^. Isotopologue replacement was achieved with a purity greater than 95%, and no ^15^N-labeled flavins were detected by mass spectrometry.

### Time-Resolved UV–Vis Spectroscopy

Absorption spectra of each protein were obtained using an Ocean Optics USB2000+ spectrometer. This instrument collects spectra from 200 to 600 nm on the millisecond timescale using a diode array detector with a minimum integration time of 10 ms. Spectra of dark-adapted YtvA, and the N94 variants, were first obtained, and then, the sample was irradiated with ∼500 mW of a 455 (±10) nm LED for ∼30 s to 1 min until the light state was generated. The light-state spectrum was then acquired immediately after the illumination was discontinued. Subsequently, spectra were recorded as a function of time during the light to dark relaxation in the absence of irradiation, and the time constant of the dark-state recovery was determined by fitting the data with the first-order exponential decay function. The measurement was acquired at 298 K.

### Steady-State FTIR Spectroscopy

Light minus dark FTIR spectra were obtained with a 1 cm^–1^ resolution on a Vertex 80v (Bruker) FTIR spectrometer using a Harrick liquid cell equipped with CaF_2_ windows and a 50 μm spacer. All samples were prepared at a 0.6^–1^ mM concentration in D_2_O buffer (20 mM Tris, 150 mM NaCl, pD 8.0). The light state was generated by 2 min irradiation using a 460 nm high-power mounted LED (Prizmatix, Ltd.) placed in the sample compartment and focused onto the cell using an objective. The temperature of the sample holder was controlled using a circulating water bath, and data were acquired at 25 °C. Two hundred fifty-six scans were acquired with a scan velocity of 20 kHz for both the dark-state and light-state spectra. The light minus dark difference spectra were generated by subtracting the light spectra from the dark spectra using OPUS 7.0.

### Time-Resolved Multiple Probe Spectroscopy (TRMPS)

TRMPS spectra were obtained at 20 °C from 100 fs to 200 μs at the STFC Central Laser Facility using a 450 nm pump operated at 0.6–0.8 μJ per pulse and a repetition rate of 1 kHz. Light-sensitive samples were analyzed using a rastered flow cell, and data were acquired from 1300 to 1800 cm^–1^ at a resolution of 3 cm^–1^ per pixel. Data were obtained by using a 50 μm path length flow cell operated at 1.5 mL/min. Pump on–pump off difference spectra were generated and converted to OD units. After the measurements were recorded, the extent of photoconversion was shown to be negligible by using absorbance spectroscopy. The spectral resolution was 3 cm^–1^, and the temporal resolution was 200 fs. A typical measurement was acquired during 45 min of data collection. All samples were prepared at a 0.6–1 mM concentration in D_2_O buffer (20 mM Tris, 150 mM NaCl, pD 8.0). Spectra were calibrated relative to the IR transmission of a pure polystyrene standard sample placed at the sample position.^51^ Data were analyzed globally using the sequential model with the Glotaran software package.^25^

### ADP^2^ Transcreener Fluorescent Polarization (FP) Assay

The production of ADP in the YF1/FixJ system was measured using a Transcreener ADP^2^ FP assay kit (3010-1K, BellBrook Laboratories). The 10 μM ADP detection mixture was prepared as described and contained 0.5× Stop&Dect Buffer B, 400 nM ADP Alexa Fluor 633 Tracer, and 11.8 μg/mL ADP^2^ antibody. The 10 μM ADP standard curve was generated by mixing 10 μL of the detection mixture with 10 μL of the appropriate concentration of the ADP/ATP mixture from 0 to 10 μM. Enzyme titration was performed with varying concentrations of YF1 and FixJ at 25 °C in the dark using 50 mM Tris–HCl reaction buffer pH 8.0 containing 50 mM KCl, 100 μM MnCl_2_, and 5% (v/v) ethylene glycol. The fluorescence polarization obtained as a function of enzyme concentrations was fitted to a nonlinear regression model using GraphPad Prism to generate the standard curve. The phosphorylation reaction was performed in the same reaction buffer in the dark or using constant illumination with a 450 nm LED. The reaction was initiated by adding 125 nM of wild-type or mutant YF1 into solutions containing 10 μM ATP and 625 nM FixJ in a 384-well low-volume round-bottom assay plate (Corning) to a final volume of 10 μL. Subsequently, 10 μL of the detection mixture was added to quench the reaction at various times after initiating the reaction to give a total of 10 time points. The change in fluorescence polarization (mP) was determined using a plate reader with 620 nm excitation and 680 nm emission (BioTek). The product formation plot was generated by converting the mP value to the ADP concentration at each time point using the standard curve. The data were fitted to a simple linear regression model using GraphPad Prism, and the initial velocity of each reaction was determined from the slope of the line.

## Supporting Information Available

TRMPS spectra of ^15^N-labeled N94D YtvA, table of IR assignments, dark- and light-state UV–vis absorbance spectra of wild-type and mutant YtvA, steady-state FTIR spectra of wild-type YtvA, wild-type YF1 and mutant YF1, and kinetic data for wild-type and mutant YF1 (PDF)

## ABBREVIATIONS

LOV: light-oxygen-voltage
STAS: sulfate transporter and anti-σ factor antagonist
PAS: Per–ARNT–Sim
TRMPS: time-resolved multiple probe spectroscopy
FP: fluorescent polarization
MD: molecular dynamics
FMN: flavin mononucleotide
EADS: evolution-associated difference spectra
EAS: evolution-associated spectra
