# Supplementary material for: Elucidating the Signal Transduction Mechanism of the Blue-Light-Regulated Photoreceptor YtvA: From Photoactivation to Downstream Regulation
Source: ACS Chem Biol. 2024 Feb 22;19(3):696–706. doi: 10.1021/acschembio.3c00722 (PMC10949197; doi:10.1021/acschembio.3c00722)
Supplement: Supplementary file 1 — cb3c00722_si_001.pdf [file cb3c00722_si_001.pdf]

Supporting Information

Elucidating the Signal Transduction Mechanism of the Blue-Light Regulated Photoreceptor YtvA: From Photoactivation to Downstream Regulation

YongLe He[†], Jinnette Tolentino Collado[†], James N. Iuliano[†], Helena A. Woroniecka[†], Christopher R. Hall[‖], Agnieszka A. Gil[†], Sergey P. Laptenok[‡], Gregory M. Greetham[‖], Boris Illarionov[∞], Adelbert Bacher[∞,ǂ], Markus Fischer[∞], Jarrod B. French[†,#], Andras Lukacs[‡,§,*], Stephen R. Meech[‡,*], and Peter J. Tonge[†,*]

[†]Department of Chemistry, Stony Brook University, Stony Brook NY, 11794, United States, [‡]School of Chemistry, University of East Anglia, Norwich, NR4 7TJ, U.K, [§]Department of Biophysics, Medical School, University of Pecs, Szigeti ut 12, 7624 Pecs, Hungary, [‖]Central Laser Facility, Research Complex at Harwell, Rutherford Appleton Laboratory, Didcot, OX11 0QX, U.K, [∞]Institut für Biochemie und Lebensmittelchemie, Universität Hamburg, Grindelallee 117, D-20146 Hamburg, Germany, [ǂ]TUM School of Natural Sciences, Technical University of Munich, 85747 Garching, Germany, [#]The Hormel Institute, University of Minnesota, Austin MN, 55912, United States

*Authors to whom correspondence should be addressed: Email: andras.lukacs@aok.pte.hu (AL); s.meech@uea.ac.uk (SRM); peter.tonge@stonybrook.edu (PJT)

Table of Contents

Table S1: IR assignment from the ^{15}N labeling spectra S3

Figure S1: The LOV domain photocycle S4

Figure S2: Impact of ^{15}N labeling on the TRMPS spectra of N94D YtvA. S5

Figure S3: Absorbance spectra and dark state recovery kinetics. S6

Figure S4: Steady-state FTIR light-dark difference spectra of wild-type and mutant YtvA S7

Figure S5: Steady-state FTIR light-dark difference spectra of YtvA and YF1 S8

Figure S6: Fluorescence polarization (FP) assay. S9

Table S1: IR assignments from the ^{15}N labeled spectra		
**Mode /cm^{-1}**	**Excited State**	**Assignments**
1537	1FMN*	N5=C4a-C10a=N1
1575	1FMN*	C4a=N5 stretch
1663	1FMN*	FMN and protein
1650	1FMN*	C2=O
1629	1FMN*	N94/N104
1616	1FMN*	N94/N104
1581	1FMN*	C4a=N5 stretch
1663	3FMN*	FMN and protein
1430	A390	Protein (proline and β-sheet)
1443	A390	Protein (proline and β-sheet)
1526	A390	N-H bent
1544/1551	A390	FMN-C4-C10a
1630- 1638	A390	FMN and protein

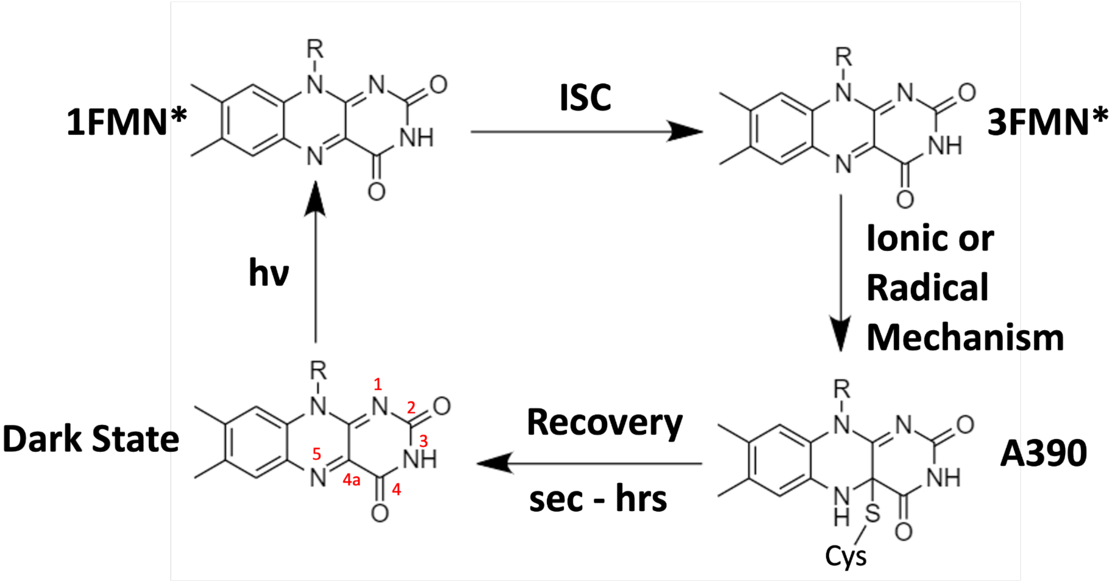

Figure S1: **The LOV domain photocycle.** The photocycle involves photoexcitation to the singlet state (1FMN*) state, followed by formation of the triplet state (3FMN*), and then the adduct state (A390). The adduct state returns to the dark state at variable rates (seconds to hours) depending on the specific LOV domain.

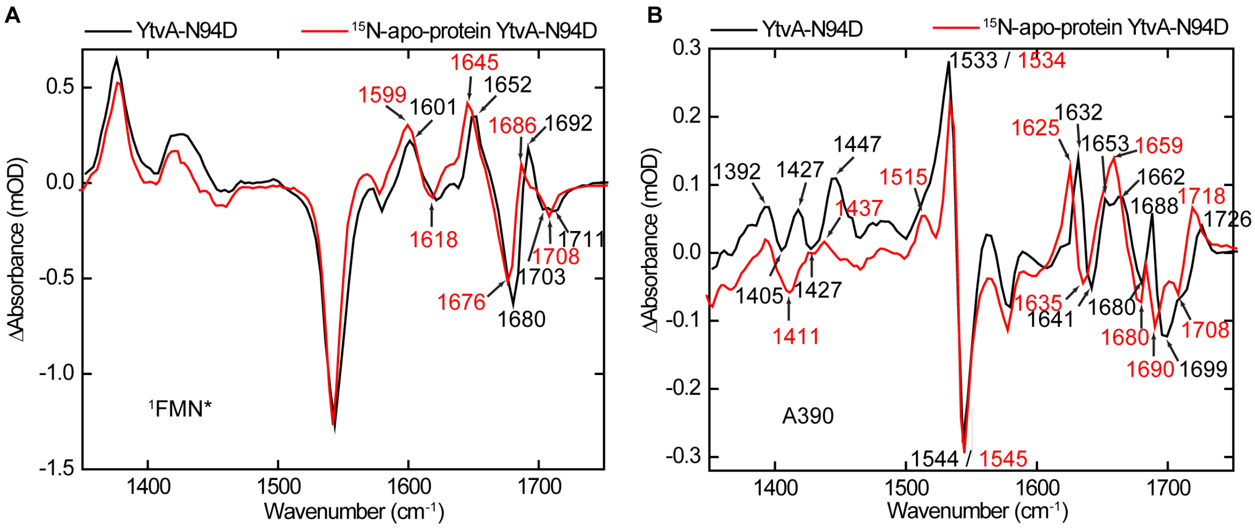

Figure S2: Impact of ^{15}N labeling on the TRMPS spectra of N94D YtvA. (A) Singlet excited state TRMPS spectra of ^{15}N labeled apoprotein containing unlabeled FMN. (B) A390 state TRIR spectra of ^{15}N labeled apoprotein containing unlabeled FMN. The graph A is the ES, graph B represents A390 state.

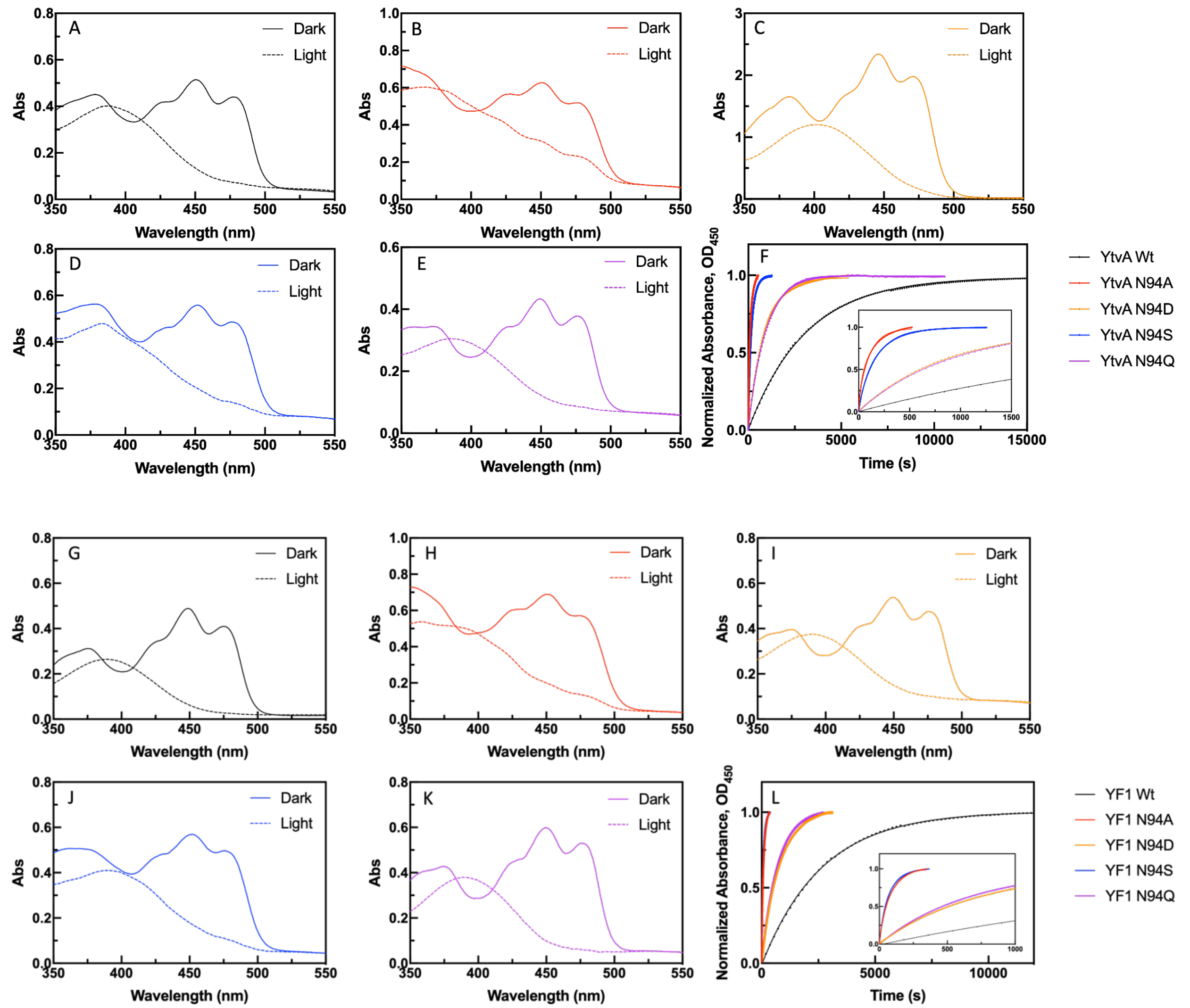

Figure S3: The UV-Vis spectra and dark state recovery kinetics of the wild-type YtvA (A), YF1(G) and N94 variants (Spectra B, C, D, E are YtvA N94A, D, S, and Q, spectra H, I, J, K are YF1 N94A, D, S and Q, respectively). Dark state recovery kinetics were obtained by monitoring the visible absorption spectrum of the flavin at 450 nm post irradiation with 455 nm LED (F is the dark recovery of YtvA wild-type and the N94 variants, L is the dark state recovery of YF1 wild-type and N94 variants). The graphs contain data from a single replicate. The experiment was repeated twice and the reported dark state recovery kinetics in Table 1 are the standard deviation of the mean.

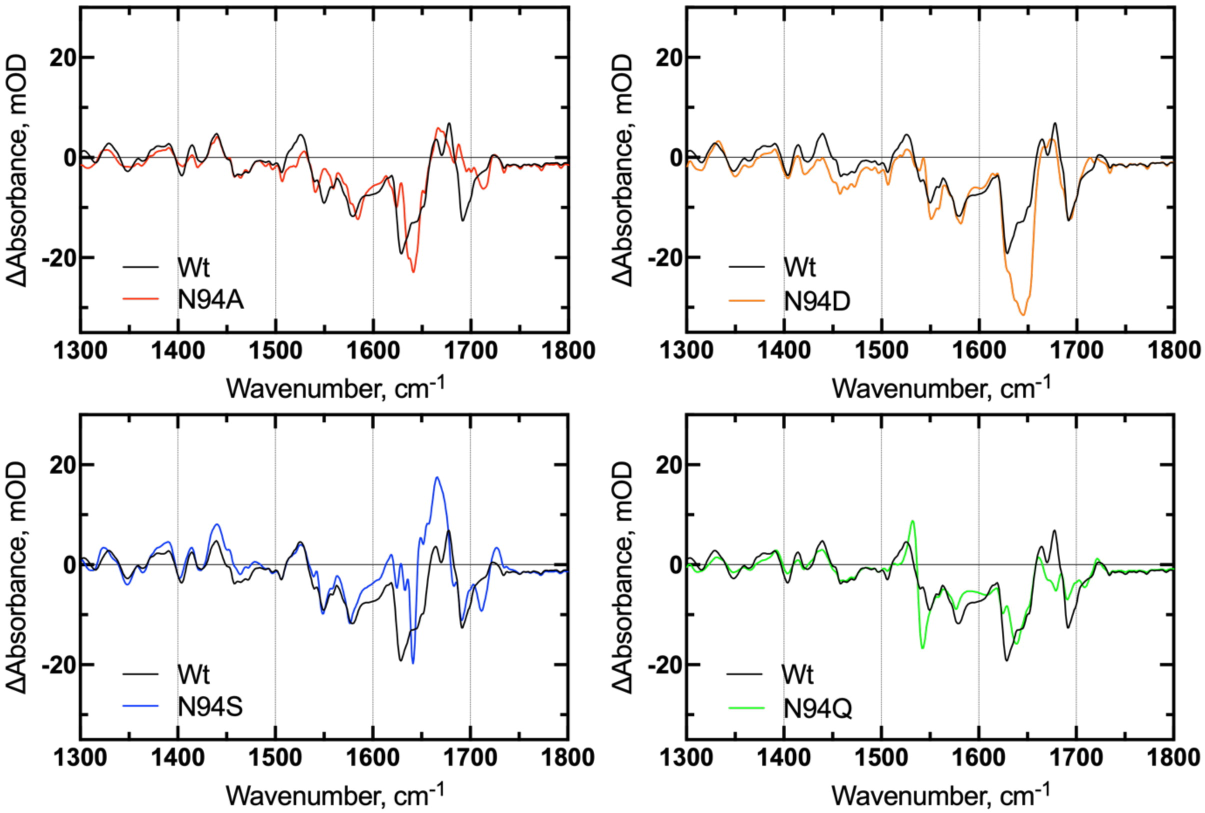

Figure S4: Steady-state FTIR light-dark difference spectra of wild-type and mutant YF1. FTIR spectra of wild-type YF1 compared with (A) N94A, (B) N94D, (C) N94S and (D) N94Q.

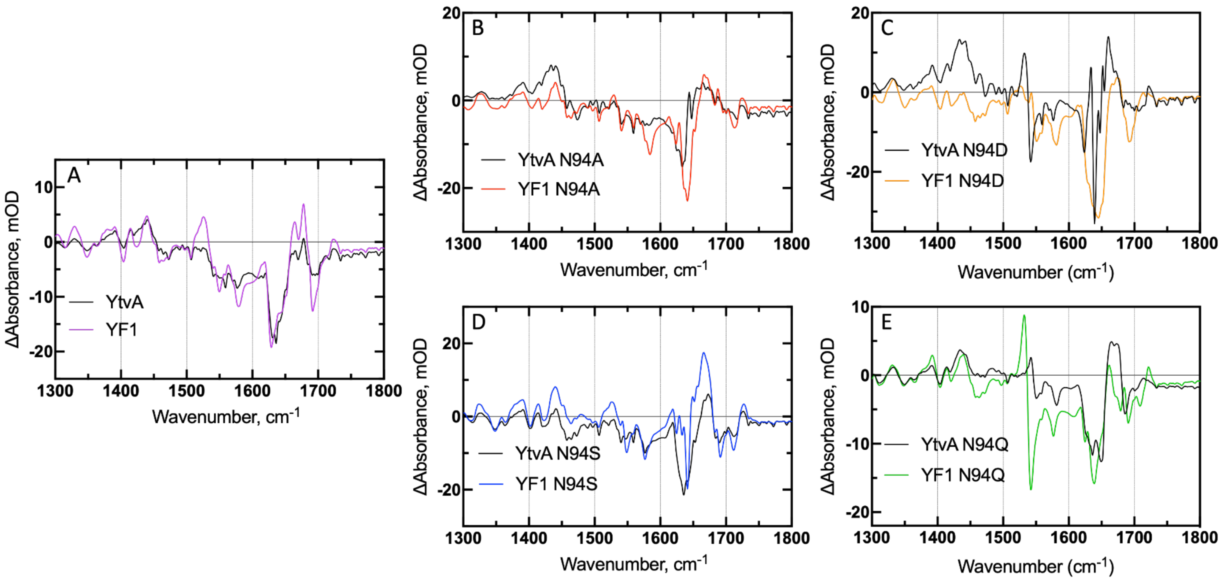

Figure S5: Steady-State FTIR light-dark difference spectra of YtvA and YF1. Comparison of the FTIR spectra of YtvA and YF1 for (A) wild-type, (B) N94A, (C) N94D, (D) N94S, and (E) N94Q.

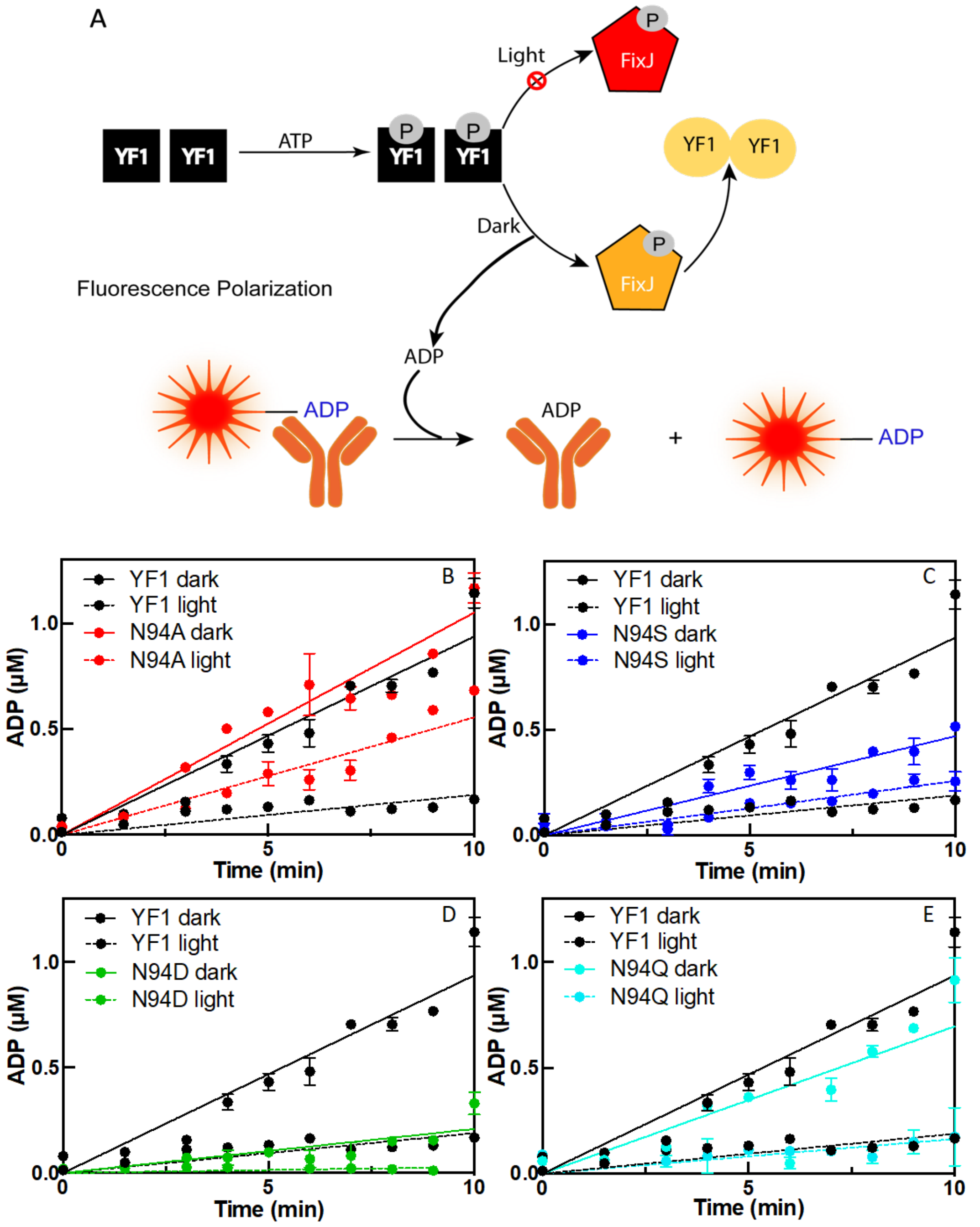

Figure S6: **Fluorescence polarization (FP) assay.** (A) Schematic diagram of the FP assay in which kinase activity is turned on in the dark and turned off by illumination. (B) to (E) The phosphorylation activity of the YF1 and the N94 variants in the presence of blue light. Wildtype YF1 exhibits kinase activity in the absence of blue light which is suppressed in the presence of blue light. The graphs contain data from duplicates and the reported error is the standard deviation of the mean.
